# Clinical Application of Radioembolization in Hepatic Malignancies: Protocol for a Prospective Multicenter Observational Study

**DOI:** 10.2196/16296

**Published:** 2020-04-22

**Authors:** Thomas Helmberger, Dirk Arnold, José I Bilbao, Niels de Jong, Geert Maleux, Anders Nordlund, Bora Peynircioglu, Bruno Sangro, Ricky A Sharma, Agnes Walk

**Affiliations:** 1 Department of Radiology, Neuroradiology and Minimal-Invasive Therapy Klinikum Bogenhausen München Germany; 2 Oncology and Hematology Asklepios Tumorzentrum Hamburg Asklepios Klinik Altona Hamburg Germany; 3 Interventional Radiology Clinica Universidad de Navarra Pamplona Spain; 4 Cardiovascular and Interventional Radiological Society of Europe Vienna Austria; 5 Radiologie Universitair Ziekenhuis Leuven Leuven Belgium; 6 Trial Form Support Aktiebolag Lund Sweden; 7 Department of Radiology School of Medicine Hacettepe University Ankara Turkey; 8 Liver Unit Clínica Universidad de Navarra Pamplona Spain; 9 Instituto de Investigación Sanitaria de Navarra Pamplona Spain; 10 Centro de Investigación Biomédica en Red Enfermedades Hepáticas y Digestivas Pamplona Spain; 11 National Institute for Health Research University College London Hospitals Biomedical Research Centre University College London Cancer Institute University College London London United Kingdom

**Keywords:** hepatocellular carcinoma, metastasis, observational study, registries, therapeutic embolization, liver, yttrium-90, radioisotope brachytherapy

## Abstract

**Background:**

Radioembolization, also known as transarterial radioembolization or selective internal radiation therapy with yttrium-90 (90Y) resin microspheres, is an established treatment modality for patients with primary and secondary liver tumors. However, large-scale prospective observational data on the application of this treatment in a real-life clinical setting is lacking.

**Objective:**

The main objective is to collect data on the clinical application of radioembolization with 90Y resin microspheres to improve the understanding of the impact of this treatment modality in its routine practice setting.

**Methods:**

Eligible patients are 18 years or older and receiving radioembolization for primary and secondary liver tumors as part of routine practice, as well as have signed informed consent. Data is collected at baseline, directly after treatment, and at every 3-month follow-up until 24 months or study exit. The primary objective of the Cardiovascular and Interventional Radiological Society of Europe Registry for SIR-Spheres Therapy (CIRT) is to observe the clinical application of radioembolization. Secondary objectives include safety, effectiveness in terms of overall survival, progression-free survival (PFS), liver-specific PFS, imaging response, and change in quality of life.

**Results:**

Between January 2015 and December 2017, 1047 patients were included in the study. The 24-month follow-up period ended in December 2019. The first results are expected in the third quarter of 2020.

**Conclusions:**

The CIRT is the largest observational study on radioembolization to date and will provide valuable insights to the clinical application of this treatment modality and its real-life outcomes.

**Trial Registration:**

ClinicalTrials.gov NCT02305459; https://clinicaltrials.gov/ct2/show/NCT02305459

**International Registered Report Identifier (IRRID):**

DERR1-10.2196/16296

## Introduction

Primary hepatic malignancies are among the most common cancers of solid organs worldwide and are the fourth to fifth leading cause of death. From the primary liver diseases cirrhosis and hepatocellular carcinoma (HCC), about 180,000 and 75,000 patients, respectively, will die per year in Europe [1,2]. Significant rises in incidence rates for hepatic inflammation, fibrosis, and cirrhosis—predisposing factors for HCC—are expected for the next decade with causes such as autoimmune disease, drug-related effects, or nonalcoholic fatty liver disease [2]. About 4 million people per year are affected by cancer in Europe; 30% to 80% of these patients might develop hepatic metastases [3].

Curative treatment of a hepatic malignancy by liver transplantation, resection, and local ablation can be applied in only about 10% to 25% of cases. Unfortunately, the vast majority of patients do not qualify for these therapies. Various kinds of systemic treatments including chemotherapy, biological therapy, and cancer immunotherapy are offered to this group of patients [4-7]. There is a substantial subset of patients with liver-limited disease that are not suitable for surgical or percutaneous ablative therapies, who experienced early recurrences or no response, significant side effects, or intolerance when treated with systemic therapies. In this setting, transarterial therapies such as chemoembolization, chemoperfusion, or radioembolization (eg, selective internal radiation therapy or transarterial radioembolization) may offer substantial therapeutic improvement [4,7]. Furthermore, these therapies may allow a reduction of systemic side effects, extend periods of freedom from chemotherapy, and prime the liver for other potential local treatment options such as surgery or local ablation [8-11].

Several current, large-scale randomized controlled trials (RCTs) could define the role of radioembolization in first-line and second-line therapy regimens in more advanced primary and secondary liver malignancies [12-15]. Based on this data, current guidelines suggest radioembolization should be considered as a component of the “tool-box” [5,6] in the treatment of HCC, cholangiocarcinoma, and metastatic colorectal cancer (mCRC) following progression after standard therapies or intolerance to other therapies [4,16].

Nevertheless, there is still limited information and understanding of the application of radioembolization in a real-life clinical setting [13,17]. Recent observational studies conducted in the United Kingdom describe the outcome of the real-life application of radioembolization in patients with colorectal liver metastases and intrahepatic cholangiocarcinoma [18,19]. However, similar real-world data from other countries and from patients with other liver malignancies such as HCC are needed by physicians, patients, and paying bodies. More observational data on the use of radioembolization in various clinical settings would elucidate the position of radioembolization in clinical practice and additionally provide data for less established uses of radioembolization, as in metastatic liver disease from other primaries (eg, breast cancer, malignant melanoma, or pancreatic cancer) [20,21].

To further improve the understanding of the real-life clinical application of radioembolization and its impact on clinical practice, the Cardiovascular and Interventional Radiological Society of Europe (CIRSE) initiated the *CIRSE Registry for SIR-Spheres Therapy (CIRT)* for patients treated with radioembolization with yttrium-90 (90Y) resin microspheres (SIR-Spheres, Sirtex Medical Pty Limited; St. Leonards, NSW, Australia). CIRT will collect data on how radioembolization is embedded in real-life clinical practice as well as effectiveness, safety, technical considerations, and patient-reported quality of life (QOL).

## Methods

### Study Design and Objectives

CIRT is a prospective, multicenter, single-device, noninterventional study of patients with liver tumors treated with radioembolization in daily routine practice as a standard of care. The primary objective is to observe the real-life clinical application of radioembolization and the impact of the treatment in clinical practice. This objective is described by: type of liver cancer, intention of treatment, prior hepatic procedures, associated systemic therapy, and postradioembolization hepatic procedures.

Secondary objectives are effectiveness, safety, technical considerations, and patient-reported outcome measures (see Textbox 1).

**Secondary end points.**

**Effectiveness end points**

- Overall survival
- Progression-free survival
- Hepatic progression-free survival (ie, liver-specific progression-free survival)
- Imaging response

**Safety end points**

- Day-of-treatment complications
- Adverse events
- Laboratory values

**Technical considerations end points**

- Patient-related characteristics
- Treatment-related characteristics
- Treatment administration
- Procedure-related outcomes

**Patient-reported outcome end points**

- Quality of life questionnaire C30
- Additional hepatocellular carcinoma module for patients with hepatocellular carcinoma

### Site Selection and Patient Enrollment

Sites that incorporated radioembolization in their standard of care armamentarium and had a minimum amount of experience with the procedures were considered for invitations to participate in the study (ie, 10 or more treatments in the last 12 months and a career history of at least 40 cases). The selected sites were limited to centers in the European Union, Switzerland, Turkey, and Israel. A multidisciplinary steering committee containing experts from the field of medical oncology, diagnostic and interventional radiology, hepatology, surgery, and nuclear medicine developed a list of sites that met the selection criteria and continued to update this list throughout the course of the study. All sites that met the selection criteria were invited directly by the steering committee.

Eligible patients consist of any adult patients treated with radioembolization with 90Y resin microspheres for primary or secondary liver tumors that have signed the informed consent form. No formal sample size calculation was made. The steering committee reasoned that about 1000 patients could be recruited during a period of 3 years, and this would be sufficient to observe the real-life clinical application of radioembolization.

Site enrollment was from August 2014 until April 2017. Patients were included from January 2015 until December 2017. Follow-up data collection ended in December 2019.

This study was performed in accordance with the Declaration of Helsinki and Good Clinical Practice Guidelines. This study was approved by the local ethics committees of participating centers.

### Data Collection

**Figure 1:**
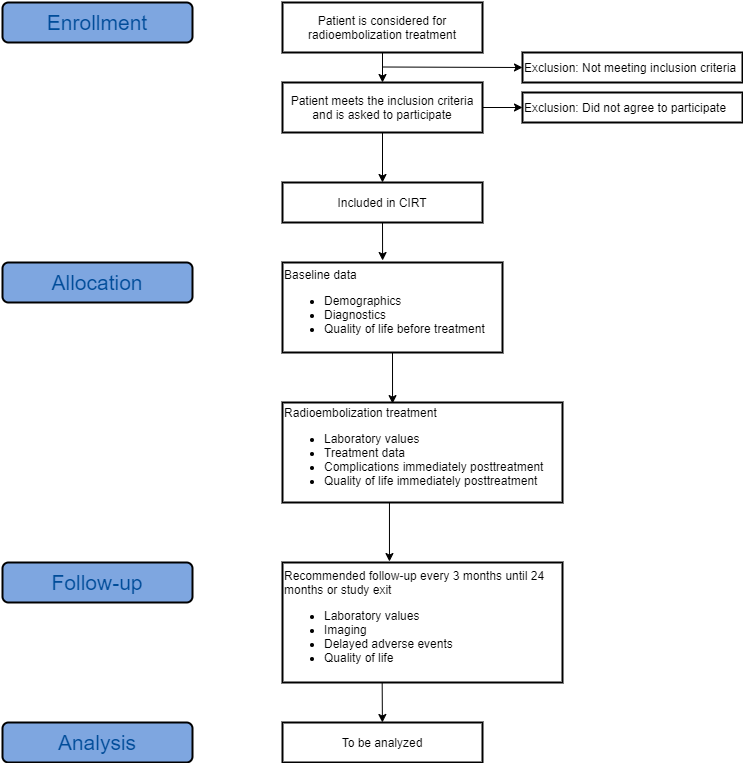
Flowchart of patient inclusion and measurement time points. CIRT: Cardiovascular and Interventional Radiological Society of Europe Registry for SIR-Spheres Therapy.

Patients enter the study by accepting participation and signing the informed consent form. Baseline data is collected upon allocation. Data on how the treatment was performed and its day-of-treatment outcomes are collected on the day of treatment. The patient is then followed for a maximum of 24 months or until study exit (see Figure 1). Guidelines for radioembolization advise that posttreatment assessments should be performed every 3 months [22]. However, the final decisions on treatment and follow-up schedules are determined by the site-specific medical teams.

Baseline assessments included patient demographics, medical history related to the underlying disease and cancer treatment, as well as cancer type and stage for patients with HCC (Table 1). The technical aspects that may determine how radioembolization is performed or impacts procedure-related outcomes were documented at the time of treatment. Time to event end points and safety end points were collected at every follow-up. In addition to demographic and clinical data, patient-reported outcomes are measured using the European Organisation for the Research and Treatment of Cancer (EORTC) quality of life questionnaire C30 (QLQ-C30) [23,24]. The questionnaire was provided before the treatment, shortly after the treatment, and at every follow-up. When this was not feasible on site, patients were approached via letters or phone calls organized by the study center responsible for the patient. For patients with HCC, in addition to the QLQ-C30, the HCC module was provided to assess factors related to chronic liver disease, as well as issues related to the primary tumor and its treatment [25]. The QLQ-C30 and HCC module were made available to each patient in their local language, using questionnaires translated and validated by EORTC [26-28].

Demographic and clinical data obtained from medical examinations, medical records, and QOL forms were transferred into an electronic case report form (e-CRF). Patient data was pseudonymized by each site without a centralized pseudonymization policy, and data collection was done according to the General Data Protection Regulation and stored in an encrypted form on a state-of-the-art server in Vienna, Austria. The e-CRF was accessible through a custom-built online database developed by ConexSys Inc (Lincoln, RI) and installed on the CIRSE server in Vienna, Austria. Access to the system is password protected. Statistical analysis will be performed by an independent statistician.

Data quality was ensured through regular remote monitoring of data entered into the database. Remote monitoring includes regular information on data quality (query reports), inclusion of patients, and follow-ups. No on-site monitoring or source data verification was performed due to limited resources.

**Table 1:** Time of measurement for each end point and associated measurements.

End point	Baseline measurement	Day of treatment measurement	Follow-up measurement
Real-life application of radioembolization	Type of liver cancer Prior hepatic procedures Associated systemic therapy (prior systemic therapy)	Intention of treatment	Postradioembolization hepatic procedures Postradioembolization systemic chemotherapy
Effectiveness end points	N/A^a^	Treatment date	Date of effectiveness event (OS^b^, PFS^c^, hepatic PFS, IR^d^)
Safety end points	N/A	Severe day of treatment complications (Grade 3-4)^e^	Adverse events (Grade 1-5)^e^ Abnormal laboratory values
Technical considerations end points	Patient-related characteristics Prior hepatic procedures Physical characteristics (body surface area, lung shunt study [%], Eastern Cooperative Oncology Group performance status)	Treatment planning Treatment administration Procedure-related outcomes	N/A
Patient-reported outcome end points	EORTC^f^ QLQ-C30^g^ Additional module for HCC^h^	N/A	EORTC QLQ-C30 Additional module for HCC

^a^N/A: not applicable.

^b^OS: overall survival.

^c^PFS: progression-free survival.

^d^IR: imaging response.

^e^Grading according to Common Terminology Criteria for Adverse Events version 4.03.

^f^EORTC: European Organisation for the Research and Treatment of Cancer.

^g^QLQ-C30: Quality of Life Questionnaire C30.

^h^HCC: hepatocellular carcinoma.

### Statistical Analysis

All patients who were found eligible, enrolled in the registry, underwent radioembolization treatment, and have the minimum amount of data required will be included in analysis. Patients for which the amount of data collected is too scarce to warrant meaningful analysis will be excluded. The number of excluded patients and reason for exclusion will be reported.

Data regarding the primary end point, safety, and technical considerations will be presented by summaries (eg, counts, means, standard deviations) and descriptive statistics. The number of missing observations will be given in all summary tables. Time to event end points will be described graphically through the Kaplan-Meier analysis (including 95% CIs for median survival). The Cox regression will be used to assess the impact of the covariates for time to event end points (Table 2).

Hazard ratios and their 95% CIs will be presented together with *P* values. Sensitivity analyses of time to event data will be performed where censored patients with less than 6 weeks of follow-up will be included as worst case (ie, death or progression).

**Table 2:** Covariates for time to event end points.

Covariate	Variables
Age (years)	≤69 and ≥70
Sex	Male, female
Number of lines of previous chemotherapy	0, 1, 2-5, ≥6
Primary tumor in situ	Yes, no
Eastern Cooperative Oncology Group performance status	0, 1, and ≥2
Presence of extrahepatic metastases	Yes, no
Prior liver procedures	Yes, no
Number of liver tumors	1, 2-5, and ≥6
Percentage tumor to liver volume	Continuous

QOL is measured with the EORTC QLQ-C30, a questionnaire developed to assess the QOL in patients with cancer. The QLQ-C30 includes five functional scales, three symptom scales, a global health status scale, and six single items. The functional and symptom status of the QLQ-C30 will be assessed according to the scoring manual developed and validated by EORTC [29]. Patients who die during follow-up will be included in the QOL analyses until their date of death. The focus of these analyses will be on patients who are alive at the time point of analysis.

To document the safety and effectiveness of radioembolization and subsequent surgical or interventional procedures, the data will be broken down into subgroups as defined for the covariate analyses above.

Missing data can be expected in the event of a study site failing to enter certain data into the e-CRF, or a patient’s withdrawal of consent or being lost to follow-up. Given the descriptive and exploratory nature of CIRT, all available data will be used, and no imputations of missing data will be made. The amount of missing data will be summarized and the subset of patients with missing data will be compared with patients included in the analysis.

In the effectiveness analyses, patients withdrawing consent or being lost to follow-up will be censored at the last time they were observed as being alive (overall survival) or at the time of their last magnetic resonance imaging scan from which disease progression could have been determined (progression-free survival [PFS]). Patients who die during the course of their follow-up in the registry will be regarded as having disease progression in the analyses of PFS and liver-specific PFS.

Missing data for QOL will be handled in line with the suggestions in the EORTC scoring manual for QLQ-C30; the amount of and reasons for completely missing forms will be analyzed and multiple imputations will be used for a part of the completely missing forms (ie, forms considered to be missing at random or due to administrative failure) [29].

## Results

Between August 2014, and April 2017, 63 hospitals that met the selection criteria were invited, 36 (57%) of which were activated (ie, were trained, had access to database, were eligible to include patients), of which 29 (81%) enrolled patients.

A total of 1047 patients were collected during the trial period between January 1, 2015, and December 31, 2017. Follow-up data collection ended on December 31, 2019. Final results are expected to be published in 2020 or 2021.

## Discussion

### Summary

CIRT is the first European-wide prospective observational study of patients with liver tumors treated with radioembolization as the standard of care and will provide an important addition to the body of knowledge on radioembolization treatment.

Data from several large RCTs on mCRC and HCC were published while CIRT was already ongoing and more can be expected in the future [12,30,31]. However, these studies provide insufficient data with respect to “real life” clinical practice since there may be patient cohorts that benefit more from radioembolization than the patient cohorts included in these trials [32-34]. CIRT was designed to also explore patient cohorts for which less data is available. The writing group hopes that the outcomes of CIRT would provide valuable insights in these less explored patient populations to gain further insight into real world clinical practice of radioembolization. The extent to which this data relates to the overall body of data available on radioembolization will be discussed in the results publication.

### Limitations

The single-arm observational study nature of the study design implies that several limitations need to be considered. The absence of a contemporaneous comparator group may limit our interpretation of the clinical data reported. Despite this, important positive features of the study are the long-term data collection for patients included and the valuable information collected on health-related QOL. The size of the study should enhance patient selection by providing new information on the patient subgroups that benefit most from this treatment in day-to-day clinical practice.

Furthermore, selection bias could occur at several stages of the study. An element of site selection was introduced when the Steering Committee created a list of sites that met the selection criteria of minimal expertise with radioembolization treatment. The potential of patient selection bias is addressed by a contractual agreed upon prospective design, whereby sites agreed to present the possibility to participate in the CIRT to all eligible patients consecutively.

Another source for bias relates to drop-out during follow-up. The sites included in the study have been advised to continue patient follow-up at least 24 months after the first treatment. However, patients may be lost to follow-up or withdraw their consent at any time. Loss to follow-up may relate to the course of disease or site procedures; for example, when a patient is treated at a participating site but followed-up by their referring physician, sometimes in another country. It will therefore not always be possible for study sites to collect additional follow-up data and the patient will be reported as lost to follow-up. Baseline characteristics will be compared between those lost to follow-up and those who have follow-up data to assess differences with the potential to bias results. Notable differences on a background characteristic will prompt additional analyses where the sensitivity of the results to that background characteristic will be further assessed.

### Conclusion

To date, the CIRT is the largest observational study on the use of radioembolization with 90Y resin microspheres. The objective of the study was to observe the real-life clinical application of radioembolization and its impact in clinical practice. Currently, radioembolization has a position in the guidelines for the treatment of HCC, cholangiocarcinoma, and mCRC, and more data is warranted on the possibilities that this treatment has for these diseases. Furthermore, additional data is needed for the effects of other lines of therapy and treatment of liver metastatic disease from other primary sites. Observing a large study population may reveal treatment aspects that would justify a wider application of this innovative treatment.

## Abbreviations

CIRSE: Cardiovascular and Interventional Radiological Society of Europe
CIRT: Cardiovascular and Interventional Radiological Society of Europe Registry for SIR-Spheres Therapy.
e-CRF: electronic case report form
EORTC: European Organization for the Research and Treatment of Cancer
HCC: hepatocellular carcinoma
mCRC: metastatic colorectal cancer
PFS: progression-free survival
QLQ-C30: quality of life questionnaire C30
QOL: quality of life
RCT: randomized controlled trials
SIR: selective internal radiation
90Y: yttrium-90.

## References

[1] Ferlay J, Colombet M, Soerjomataram I, Mathers C, Parkin DM, Piñeros M, Znaor A, Bray F (2019). Estimating the global cancer incidence and mortality in 2018: GLOBOCAN sources and methods. Int J Cancer.

[2] Blachier M, Leleu H, Peck-Radosavljevic M, Valla D, Roudot-Thoraval F (2013). The burden of liver disease in Europe: a review of available epidemiological data. J Hepatol.

[3] de Ridder J, de Wilt JH, Simmer F, Overbeek L, Lemmens V, Nagtegaal I (2016). Incidence and origin of histologically confirmed liver metastases: an explorative case-study of 23,154 patients. Oncotarget.

[4] Van Cutsem E, Cervantes A, Adam R, Sobrero A, Van Krieken JH, Aderka D, Aranda Aguilar E, Bardelli A, Benson A, Bodoky G, Ciardiello F, D'Hoore A, Diaz-Rubio E, Douillard J, Ducreux M, Falcone A, Grothey A, Gruenberger T, Haustermans K, Heinemann V, Hoff P, Köhne C-h, Labianca R, Laurent-Puig P, Ma B, Maughan T, Muro K, Normanno N, Österlund P, Oyen WJG, Papamichael D, Pentheroudakis G, Pfeiffer P, Price TJ, Punt C, Ricke J, Roth A, Salazar R, Scheithauer W, Schmoll HJ, Tabernero J, Taïeb J, Tejpar S, Wasan H, Yoshino T, Zaanan A, Arnold D (2016). ESMO consensus guidelines for the management of patients with metastatic colorectal cancer. Ann Oncol.

[5] Valle JW, Borbath I, Khan SA, Huguet F, Gruenberger T, Arnold D, ESMO Guidelines Committee (2016). Biliary cancer: ESMO Clinical Practice Guidelines for diagnosis, treatment and follow-up. Ann Oncol.

[6] Giammarile F, Bodei L, Chiesa C, Flux G, Forrer F, Kraeber-Bodere F, Brans B, Lambert B, Konijnenberg M, Borson-Chazot F, Tennvall J, Luster M, Therapy‚ Oncology and Dosimetry Committees (2011). EANM procedure guideline for the treatment of liver cancer and liver metastases with intra-arterial radioactive compounds. Eur J Nucl Med Mol Imaging.

[7] European Association for the Study of the Liver (2018). EASL Clinical Practice Guidelines: management of hepatocellular carcinoma. J Hepatol.

[8] Garlipp B, de Baere T, Damm R, Irmscher R, van Buskirk M, Stübs P, Deschamps F, Meyer F, Seidensticker R, Mohnike K, Pech M, Amthauer H, Lippert H, Ricke J, Seidensticker M (2014). Left-liver hypertrophy after therapeutic right-liver radioembolization is substantial but less than after portal vein embolization. Hepatology.

[9] Salem R, Lewandowski RJ, Kulik L, Wang E, Riaz A, Ryu RK, Sato KT, Gupta R, Nikolaidis P, Miller FH, Yaghmai V, Ibrahim SM, Senthilnathan S, Baker T, Gates VL, Atassi B, Newman S, Memon K, Chen R, Vogelzang RL, Nemcek AA, Resnick SA, Chrisman HB, Carr J, Omary RA, Abecassis M, Benson AB, Mulcahy MF (2011). Radioembolization results in longer time-to-progression and reduced toxicity compared with chemoembolization in patients with hepatocellular carcinoma. Gastroenterology.

[10] Salem R, Gilbertsen M, Butt Z, Memon K, Vouche M, Hickey R, Baker T, Abecassis MM, Atassi R, Riaz A, Cella D, Burns JL, Ganger D, Benson AB, Mulcahy MF, Kulik L, Lewandowski R (2013). Increased quality of life among hepatocellular carcinoma patients treated with radioembolization, compared with chemoembolization. Clin Gastroenterol Hepatol.

[11] Salem R, Gordon AC, Mouli S, Hickey R, Kallini J, Gabr A, Mulcahy MF, Baker T, Abecassis M, Miller FH, Yaghmai V, Sato K, Desai K, Thornburg B, Benson AB, Rademaker A, Ganger D, Kulik L, Lewandowski RJ (2016). Y90 radioembolization significantly prolongs time to progression compared with chemoembolization in patients with hepatocellular carcinoma. Gastroenterology.

[12] Chauhan N, Mulcahy MF, Salem R, Benson Iii AB, Boucher E, Bukovcan J, Cosgrove D, Laframboise C, Lewandowski RJ, Master F, El-Rayes B, Strosberg JR, Sze DY, Sharma RA (2019). TheraSphere yttrium-90 glass microspheres combined with chemotherapy versus chemotherapy alone in second-line treatment of patients with metastatic colorectal carcinoma of the liver: protocol for the EPOCH phase 3 randomized clinical trial. JMIR Res Protoc.

[13] Sposito C, Mazzaferro V (2018). The SIRveNIB and SARAH trials, radioembolization vs sorafenib in advanced HCC patients: reasons for a failure, and perspectives for the future. Hepatobiliary Surg Nutr.

[14] Vilgrain V, Pereira H, Assenat E, Guiu B, Ilonca AD, Pageaux G, Sibert A, Bouattour M, Lebtahi R, Allaham W, Barraud H, Laurent V, Mathias E, Bronowicki J, Tasu J, Perdrisot R, Silvain C, Gerolami R, Mundler O, Seitz J, Vidal V, Aubé C, Oberti F, Couturier O, Brenot-Rossi I, Raoul J, Sarran A, Costentin C, Itti E, Luciani A, Adam R, Lewin M, Samuel D, Ronot M, Dinut A, Castera L, Chatellier G, SARAH Trial Group (2017). Efficacy and safety of selective internal radiotherapy with yttrium-90 resin microspheres compared with sorafenib in locally advanced and inoperable hepatocellular carcinoma (SARAH): an open-label randomised controlled phase 3 trial. Lancet Oncol.

[15] Wasan HS, Gibbs P, Sharma NK, Taieb J, Heinemann V, Ricke J, Peeters M, Findlay M, Weaver A, Mills J, Wilson C, Adams R, Francis A, Moschandreas J, Virdee PS, Dutton P, Love S, Gebski V, Gray A, van Hazel Guy, Sharma RA, FOXFIRE trial investigators, SIRFLOX trial investigators, FOXFIRE-Global trial investigators (2017). First-line selective internal radiotherapy plus chemotherapy versus chemotherapy alone in patients with liver metastases from colorectal cancer (FOXFIRE, SIRFLOX, and FOXFIRE-Global): a combined analysis of three multicentre, randomised, phase 3 trials. Lancet Oncol.

[16] European Association For The Study Of The Liver, European Organisation For Research And Treatment Of Cancer (2012). EASL-EORTC clinical practice guidelines: management of hepatocellular carcinoma. J Hepatol.

[17] van Hazel GA, Heinemann V, Sharma NK, Findlay MPN, Ricke J, Peeters M, Perez D, Robinson BA, Strickland AH, Ferguson T, Rodríguez J, Kröning H, Wolf I, Ganju V, Walpole E, Boucher E, Tichler T, Shacham-Shmueli E, Powell A, Eliadis P, Isaacs R, Price D, Moeslein F, Taieb J, Bower G, Gebski V, Van Buskirk M, Cade DN, Thurston K, Gibbs P (2016). SIRFLOX: randomized phase III trial comparing girst-line mFOLFOX6 (plus or minus bevacizumab) versus mFOLFOX6 (plus or minus bevacizumab) plus selective internal radiation therapy in patients with metastatic colorectal cancer. J Clin Oncol.

[18] White J, Carolan-Rees G, Dale M, Morgan HE, Patrick HE, See TC, Beeton EL, Swinson DEB, Bell JK, Manas DM, Crellin A, Slevin NJ, Sharma RA (2019). Analysis of a national programme for selective internal radiation therapy for colorectal cancer liver metastases. Clin Oncol (R Coll Radiol).

[19] White J, Carolan-Rees G, Dale M, Patrick HE, See TC, Bell JK, Manas DM, Crellin A, Slevin NJ, Sharma RA (2019). Yttrium-90 transarterial radioembolization for chemotherapy-refractory intrahepatic cholangiocarcinoma: a prospective, observational study. J Vasc Interv Radiol.

[20] Kuei A, Saab S, Cho S, Kee ST, Lee EW (2015). Effects of Yttrium-90 selective internal radiation therapy on non-conventional liver tumors. World J Gastroenterol.

[21] Barbier CE, Garske-Román U, Sandström M, Nyman R, Granberg D (2016). Selective internal radiation therapy in patients with progressive neuroendocrine liver metastases. Eur J Nucl Med Mol Imaging.

[22] Mahnken AH, Spreafico C, Maleux G, Helmberger T, Jakobs TF (2013). Standards of practice in transarterial radioembolization. Cardiovasc Intervent Radiol.

[23] Aaronson NK, Ahmedzai S, Bergman B, Bullinger M, Cull A, Duez NJ, Filiberti A, Flechtner H, Fleishman SB, de Haes JC (1993). The European Organization for Research and Treatment of Cancer QLQ-C30: a quality-of-life instrument for use in international clinical trials in oncology. J Natl Cancer Inst.

[24] Fayers P, Bottomley A, EORTC Quality of Life Group, Quality of Life Unit (2002). Quality of life research within the EORTC-the EORTC QLQ-C30. Eur J Cancer.

[25] Blazeby JM, Currie E, Zee BCY, Chie W, Poon RT, Garden OJ, EORTC Quality of Life Group (2004). Development of a questionnaire module to supplement the EORTC QLQ-C30 to assess quality of life in patients with hepatocellular carcinoma, the EORTC QLQ-HCC18. Eur J Cancer.

[26] Scott NW, Etta JA, Aaronson NK, Bottomley A, Fayers PM, Groenvold M, Koller M, Kuliś D, Marais D, Petersen MA, Sprangers MAG (2013). An evaluation of the response category translations of the EORTC QLQ-C30 questionnaire. Qual Life Res.

[27] Scott NW, Fayers PM, Bottomley A, Aaronson NK, de Graeff A, Groenvold M, Koller M, Petersen MA, Sprangers MAG, EORTC and the Quality of Life Cross-Cultural Meta-Analysis Group (2006). Comparing translations of the EORTC QLQ-C30 using differential item functioning analyses. Qual Life Res.

[28] Cankurtaran ES, Ozalp E, Soygur H, Ozer S, Akbiyik DI, Bottomley A (2008). Understanding the reliability and validity of the EORTC QLQ-C30 in Turkish cancer patients. Eur J Cancer Care (Engl).

[29] Fayers P, Aaronson N, Bjordal K, Groenvold M, Curran D, Bottomley A (2001). The EORTC QLQ-C30 Scoring Manual (3rd Edition).

[30] Chauhan N, Bukovcan J, Boucher E, Cosgrove D, Edeline J, Hamilton B, Kulik L, Master F, Salem R (2018). Intra-Arterial TheraSphere Yttrium-90 Glass Microspheres in the Treatment of Patients With Unresectable Hepatocellular Carcinoma: Protocol for the STOP-HCC Phase 3 Randomized Controlled Trial. JMIR Res Protoc.

[31] Gebski V, Gibbs E, Gandhi M, Chatellier G, Dinut A, Pereira H, Chow PK, Vilgrain V (2017). VESPRO: an individual patient data prospective meta-analysis of selective internal radiation therapy versus sorafenib for advanced, locally advanced, or tecurrent hepatocellular carcinoma of the SARAH and SIRveNIB Trials. JMIR Res Protoc.

[32] Bester L, Meteling B, Pocock N, Pavlakis N, Chua TC, Saxena A, Morris DL (2012). Radioembolization versus standard care of hepatic metastases: comparative retrospective cohort study of survival outcomes and adverse events in salvage patients. J Vasc Interv Radiol.

[33] Seidensticker R, Denecke T, Kraus P, Seidensticker M, Mohnike K, Fahlke J, Kettner E, Hildebrandt B, Dudeck O, Pech M, Amthauer H, Ricke J (2012). Matched-pair comparison of radioembolization plus best supportive care versus best supportive care alone for chemotherapy refractory liver-dominant colorectal metastases. Cardiovasc Intervent Radiol.

[34] Hendlisz A, Van den Eynde M, Peeters M, Maleux G, Lambert B, Vannoote J, De Keukeleire K, Verslype C, Defreyne L, Van Cutsem E, Delatte P, Delaunoit T, Personeni N, Paesmans M, Van Laethem J, Flamen P (2010). Phase III trial comparing protracted intravenous fluorouracil infusion alone or with yttrium-90 resin microspheres radioembolization for liver-limited metastatic colorectal cancer refractory to standard chemotherapy. J Clin Oncol.

